# HIV-1 Rev protein specifies the viral RNA export pathway by suppressing TAP/NXF1 recruitment

**DOI:** 10.1093/nar/gku304

**Published:** 2014-04-20

**Authors:** Ichiro Taniguchi, Naoto Mabuchi, Mutsuhito Ohno

**Affiliations:** Institute for Virus Research, Kyoto University, Kyoto 606-8507, Japan

## Abstract

Nuclear RNA export pathways in eukaryotes are often linked to the fate of a given RNA. Therefore, the choice of export pathway should be well-controlled to avoid an unfavorable effect on gene expression. Although some RNAs could be exported by more than one pathway, little is known about how the choice is regulated. This issue is highlighted when the human immunodeficiency virus type 1 (HIV-1) Rev protein induces the export of singly spliced and unspliced HIV-1 transcripts. How these RNAs are exported is not well understood because such transcripts should have the possibility of utilizing CRM1-dependent export via Rev or cellular TAP/NXF1-dependent export via the transcription/export (TREX) complex, or both. Here we found that Rev suppressed TAP/NXF1-dependent export of model RNA substrates that recapitulated viral transcripts. In this effect, Rev interacted with the cap-binding complex and inhibited the recruitment of the TREX complex. Thus, Rev controls the identity of the factor occupying the cap-proximal region that determines the RNA export pathway. This ribonucleoprotein remodeling activity of Rev may favor viral gene expression.

## INTRODUCTION

Different classes of RNA in eukaryotic cells are exported by distinct sets of export factors, that is, by distinct export pathways (1,2). In bulk mRNA export, a multi-protein complex, the transcription/export (TREX) complex, is recruited to a region near the 5′-terminal cap structure of mRNA since the cap-binding complex (CBC) directly interacts with Aly/REF, a component of the TREX complex (3–5). The complex in turn recruits the major mRNA export receptor TAP-p15 heterodimer to mRNAs (TAP and p15 are also called NXF1 and NXT1, respectively) (6–9) mainly through the interaction between TAP and Aly/REF (10–12). In addition to the mechanism that recruits TAP to mRNAs, the nuclear retention mechanism contributes to the fidelity of mRNA export. Intron-containing pre-mRNAs are retained in the nucleus, as a result of the formation of splicing complexes, until they are completely spliced (13,14).

**Figure 1.**
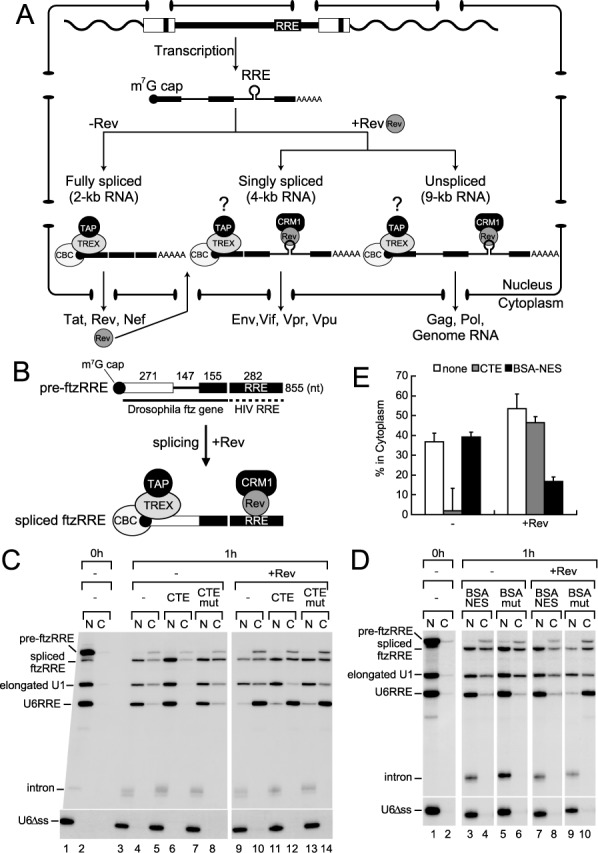
Reconstruction of the export of the singly spliced transcript in *Xenopus* oocytes. (**A**) Gene expression of HIV-1 is regulated by RNA export. (**B**) Schematic representation of pre-ftzRRE RNA used for the export analysis. (**C**) ^32^P-labeled pre-ftzRRE RNA was microinjected into the nucleus of *Xenopus* oocytes together with ^32^P-labeled U1ftz (elongated U1), U6RRE and U6Δss, in either the absence (lanes 1–8) or presence (lanes 9–14) of the purified recombinant Rev protein (160 fmol/oocyte), with either CTE (50 fmol/oocyte; lanes 5, 6, 11 and 12) or the CTE mutant M36, which does not bind TAP/NXF1 (CTEmut, 50 fmol/oocyte; lanes 7, 8, 13 and 14), or without the inhibitor (lanes 1–4, 9 and 10). RNA was extracted from nuclear (N) and cytoplasmic (C) fractions, immediately (0 h; lanes 1 and 2) or 1 h (1 h; lanes 3–14) after the injection, and were analyzed using 6% denaturing PAGE. (**D**) The same experiments as in (C) were performed in the absence (lanes 1–6) or presence (lanes 7–10) of Rev, except with BSA-NES (190 ng/oocyte; lanes 3, 4, 7 and 8) or BSA-mut (M10) NES, which does not bind CRM1 (190 ng/oocyte; lanes 5, 6, 9 and 10), or without the inhibitor (lanes 1 and 2). RNA export was analyzed immediately (0 h; lanes 1 and 2) or 1 h (1 h; lanes 3–10) after the injection. (**E**) Quantitation of the export of spliced ftzRRE RNA from three independent experiments performed as in (C) and (D). Averages and standard deviations with CTE (gray bars) or BSA-NES (black bars), or without inhibitors (none; white bars) in the absence (-) or presence (+Rev) of Rev are shown.

In some RNA viruses, such as human immunodeficiency virus type 1 (HIV-1), cellular and viral protein factors and *cis*-acting RNA elements serve to overcome the nuclear retention mechanism for intron-containing transcripts (1,15,16). HIV-1 has two intronic regions in its genome. HIV-1-infected cells produce three types of viral transcripts, an unspliced transcript containing both introns (9-kb transcript), singly spliced transcripts in which only the first intron has been removed (4-kb transcript), and fully spliced transcripts (2-kb transcript). The current consensus view of RNA export regulation by the viral protein Rev is as follows (Figure 1A): in the early stage of infection, only the fully spliced transcript is exported to the cytoplasm, presumably via the TAP-dependent mRNA export pathway. The fully spliced transcript encodes the Rev protein, which has a specific activity to bind to the Rev response element (RRE) located in the second intron. Rev has both a nuclear localization signal that is recognized by a subset of importins/karyopherins (17,18) and a leucine-rich nuclear export signal (NES) that is recognized by CRM1 (19–23); therefore, it can shuttle between the nucleus and cytoplasm through nuclear pore complexes (NPCs). In the later stage of infection, the Rev protein, which has accumulated in the nucleus, binds to RRE and promotes the export of both singly spliced and unspliced transcripts via the CRM1-dependent pathway, which leads to the expression of different sets of viral protein factors and eventually to propagation of the virus (1).

Despite the above elegant paradigm, issues regarding the HIV-1 RNA export still remain and need to be clarified. For example, if we consider how singly spliced and unspliced transcripts are actually exported, the situation is not at all simple. They should have both the TREX complex and Rev on the same RNA molecule, and, thus, have the possibility of utilizing either the TAP-dependent or CRM1-dependent export pathway, or both. This issue is important since accumulating evidence has shown that the export pathway often influences the nuclear/cytoplasmic fate of a given RNA (24–31). Therefore, the two different pathways for transcripts could have an unfavorable effect on HIV-1 gene expression. However, how HIV-1 resolves this is not understood.

## MATERIALS AND METHODS

### DNA constructs

For the pre-ftzRRE plasmid, the RRE260 fragment from pBS-RRE260 (32) was cloned into the SmaI site of pGEM-preftz (33). For the intronless ftzRRE plasmid, the intron was removed by polymerase chain reaction (PCR) from the pre-ftzRRE plasmid. U6RRE was constructed essentially as described (34). For pre-CDCRRE and pre-betaRRE, the RRE260 fragment was inserted into the KpnI site of pSP14-15 (35) and the BamHI-EcoRI sites of pSP64-HβΔ6 (36), respectively. For RRE260 and ERR350, pBS-RRE260 and pBS-ERR350 (a PCR-fragment of 350 nt RRE was cloned into the HindIII-EcoRI sites of pBS) were linearized by EcoRI and HindIII, respectively, and transcribed by T7 RNA polymerase. For pGEX-6P-1-T7-Rev, a PCR-fragment containing the full-length Rev was cloned into the EcoRI-XhoI sites of pGEX-6P-1 and annealed oligo DNAs corresponding to the T7-tag sequence were subsequently inserted into the EcoRI site. pGEX-6P-1-T7-RevM10 was constructed by site-directed mutagenesis of pGEX-6P-1-T7-Rev.

### Recombinant proteins

GST-T7-Rev or GST-T7-RevM10 was expressed from *Escherichia* *coli* BL21 (DE3) Codon Plus RIL (Stratagene) harboring pGEX-6P-1-T7-Rev or pGEX-6P-1-T7-RevM10, respectively, by the standard procedure, and the expressed protein in the bacterial lysate was bound to Glutathione Sepharose beads (GE Healthcare). The bound beads were washed three times with Buffer 1 (50 mM Tris-HCl [pH 7.5], 0.5 M NaCl, 1 mM EDTA, 1 mM DTT and 8% glycerol), and were subsequently treated with PreScission Protease (GE Healthcare; 2U/μl in Buffer 1) at 4°C for 4 days. The mixture was spun down and the supernatant was recovered as a purified fraction. FLAG-UAP56 and GST-Aly/REF (37), GST-hnRNP A1 (27), and CBC (38) were purified as previously described.

### *In vitro* transcription

^32^P-labeled RNAs were transcribed as described previously (39), except that only [α-^32^P] UTP was used. The transcription reaction was commonly performed at 37°C for 45–60 min.

### RNA microinjection into *Xenopus* oocytes

RNA microinjection into *Xenopus* oocytes was performed as previously described (39,40). To prepare BSA-NES and BSA-mut, the PKI NES peptide (CELALKLAGLDIN) or a mutant peptide (CELALKAAGADIN) was conjugated to BSA as described previously (19). Analysis and quantitation of RNA bands were performed with BAS-2500 (Fujifilm) and Image Gauge Version 3.45 (Fujifilm).

### RNA immunoprecipitation

RNA immunoprecipitation (IP) was performed as described previously (40,41). Briefly, the nuclear fraction was mixed with the indicated antibodies that had been pre-bound to Protein A-Sepharose beads (GE Healthcare), equilibrated with RSB100N buffer (10 mM Tris-HCl [pH 7.5], 100 mM NaCl, 2.5 mM MgCl_2_ and 0.1% Nonidet P-40), and rotated at 4°C for 1 h. After the beads were washed five times with RSB100N buffer, they were incubated in Homomix (50 mM Tris-HCl [pH7.5], 5 mM EDTA, 1.5% SDS, 300 mM NaCl and 1.5 mg/ml proteinase K; Nacalai Tesque) for 30 min at 50°C. RNA was recovered from the supernatants by phenol/chloroform extraction and ethanol precipitation, and analyzed by denaturing PAGE and autoradiography. IP using RNase-digested singly labeled RNA samples was performed as described (42), except that pre-ftz RRE and intronless ftzRRE RNAs were used.

### *In vitro* RNA-Rev binding assay

^32^P-labeled RNAs were mixed with purified recombinant T7-Rev in the presence of yeast tRNA (1 mg/ml) and in the presence or absence of CBC, and incubated at 30°C for 30 min. Then RNA IP was performed as above.

### GST-TAP231 pull-down and RNase H digestion/protection assay

GST-TAP231 pull-down and RNase H digestion/protection assay were performed as described previously (42,43). DNA oligos were as follows: A 5′-AGAGAGGTGGGC-3′, B 5′-CGATGTGCGACC-3′, C 5′-CTTGATCTGCCT-3′, D 5′-CCTGTACCGTCA-3′, E 5′-AATCGCCGGCTC-3′.

### Analysis of the composition of EJC

Pre-ftzRRE RNA, singly labeled near the exon–exon junction, was generated (42) and the splicing reaction was performed with this substrate in the absence or presence of Rev. The reaction mixture was treated with a mixture of RNase A and RNase T1. A short labeled RNA fragment corresponding to the exon–exon junction was protected due to exon junction complex (EJC) formation. To obtain information on the composition of EJC, IP was performed with the RNase-digested sample.

### Transfection

HEK293T cells were maintained under an atmosphere containing 5% CO_2_ in Dulbecco's modified Eagle's medium supplemented with 10% fetal bovine serum, 100 unit/ml penicillin and 100 μg/ml streptomycin. pNL4-3ΔRev (a kind gift from Drs M. Matsuoka and E. Kodama), pCI-FLAG-Rev (44), or pcDNA5-FLAG-TAP (27), pcDNA5-FLAG-p15 and pcDNA3-GFP (45) were transfected with polyethyleneimine into 70% confluent HEK293T cells in a 6-well plate. After 24 h, the cells were harvested and washed with phosphate buffered saline (PBS). The cell pellets were used for RNA and protein analyses. The culture supernatants (2 ml of culture equivalent) were filtrated through 0.45 μm filters. Samples were immunoprecipitated with anti-HIV-1 p55 antiserum raised in rabbits (Bio Academia), and the IPed proteins were subjected to western blotting using a murine anti-HIV-1 p24 monoclonal antibody (Abcam).

### Semi-quantitative RT-PCR

Total RNA was prepared from culture cells with Sepasol-RNA I Super (Nacalai Tesque) according to the manufacturer's instructions. RNAs were subsequently treated with RNase-free DNase (RQ1; Promega) according to the manufacturer's manual. First-strand cDNA was synthesized using reverse transcription (SuperScript III First-Strand Synthesis System; Life Technologies) with a random hexamer, and amplified by PCR using an appropriate set of primers. PCR products were separated by 2% agarose gel electrophoresis and stained with SYBR Gold (Life Technologies). Images were obtained with an imaging analyzer (Typhoon; GE healthcare). Primers for PCR were as follows (46):

unspliced transcript forward 5′-TTCTTCAGAGCAGACCAGAGCC-3′,
unspliced transcript reverse 5′-CGCTGCCAAAGAGTGATCTGAG-3′,
singly spliced transcript forward 5′-GCGGCGACTG GAAGAAGC-3′,
singly spliced transcript reverse 5′-CTCATTGCCACTGTCTTCTGCTC-3′,
fully spliced transcript forward 5′-GCGGCGACTGAATTGGGTGT-3′,
fully spliced transcript reverse 5′-GATTGGGAGGTGGGT TGCTTTG-3′,
GFP mRNA forward 5′-AACCACTACCTGAGCACCCAG-3′,
GFP mRNA reverse 5′-CACCACACTGGACTAGTGGATC-3′.

### GST pull-down

Purified recombinant CBP80 (3 μg) was incubated with either GST, GST-T7-Rev, GST-Aly/REF or GST-hnRNP A1 (1 μg) in the presence of 1 mg/ml RNase A and in the presence or absence of T7-Rev (0, 2.5, 7.5, 25 μg) in 20 μl D’K100 buffer (20 mM Hepes-KOH [pH 7.9], 100 mM KCl, 0.2 mM EDTA, 1 mM DTT and 10% glycerol) at 30°C for 30 min. The reaction mixture was rotated with Glutathione Sepharose beads in 100 μl RSB100N at 4°C for 1 h. After washing five times with RSB100N, the bound material was recovered and analyzed by SDS-PAGE/western blotting.

## RESULTS

### Recapitulation of the export of the singly spliced HIV-1 transcript in *Xenopus* oocytes

Elucidating the framework of Rev function was greatly aided by experiments using the heterologous *Xenopus* oocyte microinjection system, in which RNA export can be separated from other steps of gene expression, making it possible to clearly demonstrate the function of Rev in RNA export (19,34,47,48). To understand the nuclear export of HIV-1 transcripts, we first sought to reconstruct the export of singly spliced transcripts in the well-established *Xenopus* oocyte microinjection system, and generated an artificial RNA substrate, pre-ftzRRE, schematically shown in Figure 1B. The RRE sequence from HIV-1 was fused to the *Drosophila fushitarazu* (ftz) gene containing an intron, one of the most conventional splicing substrates used in *Xenopus* oocytes and mammalian cells (29,49). The spliced product of pre-ftzRRE RNA may have both the TREX complex and Rev on the same RNA in cases in which the Rev protein was supplied (Figure 1B). By which pathway this spliced RNA was exported was examined in the *Xenopus* oocyte microinjection experiments.

After 1 h incubation, pre-ftzRRE RNA had been efficiently spliced, and a fraction of spliced ftzRRE RNA had been exported to the cytoplasm, while the excised intron stayed in the nucleus (Figure 1C, lanes 3 and 4). Elongated U1-ftz RNA (elongated U1 RNA), which was previously shown to behave like an mRNA in nuclear export (29,41), was also partially exported, whereas U6RRE (34) and U6Δss RNAs mostly or completely stayed in the nucleus, respectively (Figure 1C, lanes 3 and 4). When the same RNA mixture was injected with the purified recombinant Rev protein, the export of U6RRE was greatly stimulated, as expected (34), while export of the elongated U1 was hardly affected. The export of spliced ftzRRE RNA was slightly stimulated (Figure 1C, lanes 9 and 10, and E for quantitation).

Two inhibitors of RNA export were employed to examine the export pathway of spliced ftzRRE in the absence or presence of the Rev protein. The first was constitutive transport element (CTE) RNA from type D retroviruses, which was shown to specifically bind to TAP and thereby inhibit mRNA export when a saturating amount was injected (50,51). The second was a conjugate of NES peptides coupled to BSA (BSA-NES), which was shown to saturate CRM1-dependent export (19).

When a saturating amount of CTE was injected together with RNAs in the absence of the Rev protein, the export of spliced ftzRRE RNA and elongated U1 RNA was inhibited as expected, which indicated that these RNAs utilized the mRNA pathway (Figure 1C, lanes 5 and 6, and E). When BSA-NES was injected in the absence of Rev, the export of spliced ftzRRE RNA and elongated U1 RNA was consistently unaffected (Figure 1D, lanes 3 and 4, and E). In contrast, in the presence of Rev, the export of U6RRE was not affected by CTE (Figure 1C, lanes 11 and 12), but was severely inhibited by BSA-NES (Figure 1D, lanes 7 and 8), indicating that U6RRE utilized the CRM1-dependent pathway in the presence of Rev. Most importantly, the export of spliced ftzRRE RNA was also unaffected by CTE (Figure 1C, lanes 11 and 12), but was strongly inhibited by BSA-NES (Figure 1D, lanes 7 and 8), which indicated that the majority of spliced ftzRRE RNA was exported via the CRM1-dependent pathway in the presence of Rev. These results clearly indicated that the export of spliced ftzRRE RNA almost exclusively utilized the CRM1-dependent pathway in the presence of Rev. Since the accumulation in the cytoplasm of the singly spliced transcript was shown to be dependent on Rev activity in HIV-1-infected cells (52,53), it is very likely that the above model system in *Xenopus* oocytes recapitulated the export of the singly spliced HIV-1 transcript.

### HIV-1 Rev not only induces CRM1-dependent export, but also inhibits TAP-dependent export of RRE-containing spliced RNAs

**Figure 2.**
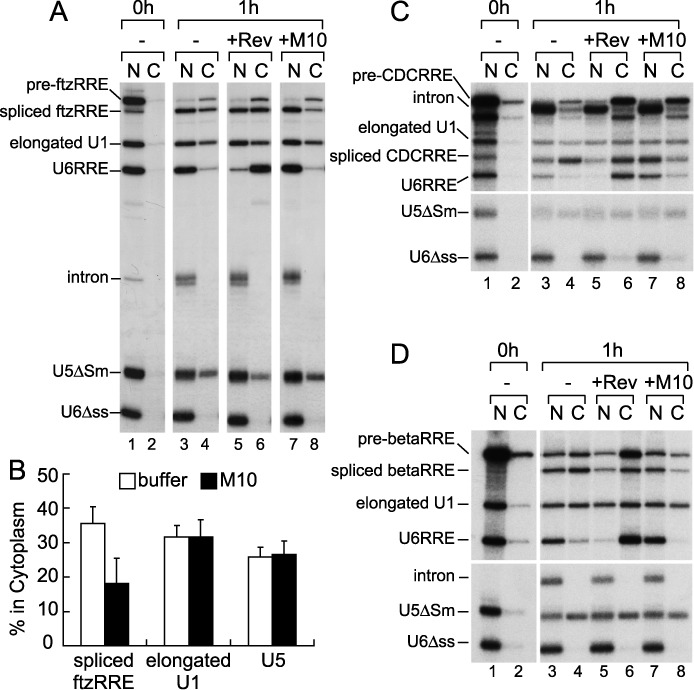
Inhibition of TAP-dependent RNA export by Rev. (**A**) The same ^32^P-labeled RNA mixture as in Figure 1 together with ^32^P-labeled m^7^G-capped U5ΔSm RNA was microinjected into the nucleus of oocytes. The effect of the wild-type Rev or Rev M10 mutant protein (160 fmol/oocyte) was examined as in Figure 1. (**B**) Quantitation of the export of spliced ftzRRE, elongated U1 and U5 RNAs from three independent experiments performed as in (A). Averages and standard deviations with M10 (black bars) or without proteins (buffer; white bars) are shown. (**C** and **D**) The same ^32^P-labeled RNA mixture as in (A), except that either pre-CDCRRE (C) or pre-betaRRE (D) was used instead of pre-ftzRRE, was microinjected into the nucleus. The effect of the wild-type Rev or Rev M10 mutant protein (160 fmol/oocyte) was examined as in (A). See also Supplementary Figure S1 for quantitation.

The export pathway of spliced ftzRRE RNA was almost completely shifted from the TAP-dependent mRNA pathway to the CRM1-dependent pathway by the presence of Rev. One might think that RNA could be exported by the TAP-dependent pathway similar to elongated U1 RNA in the presence of both Rev and BSA-NES; however, the export of RNA was actually inhibited (Figure 1E, black bar). This strongly suggested that the Rev protein prevented spliced ftzRRE RNA from utilizing the TAP-dependent export pathway. To confirm this, we next examined the effect of the Rev M10 mutant on RNA export (Figure 2). Since M10 has a mutation in the NES, it is unable to interact with CRM1 (20,54). If the Rev M10 mutant also inhibits the TAP-dependent export of spliced ftzRRE RNA, the inhibitory activity of Rev can be clearly separated from its activity to induce CRM1-dependent export.

Co-injection of the wild-type Rev protein had the same effect as described above. However, co-injection of the same amount of the M10 protein markedly inhibited the export of spliced ftzRRE RNA relative to that with the buffer control, whereas export of the other RNAs was unaffected (Figure 2A, lanes 3, 4, 7 and 8, and B for quantitation). When we employed pre-ftzERR RNA, in which the RRE sequence was fused to ftz pre-mRNA in the antisense orientation, the M10 protein had no effect (Supplementary Figure S1A and B). Moreover, when we employed pre-CDCRRE and pre-betaRRE, in which RRE was fused to chicken δ-crystallin pre-mRNA (35) and human β-globin pre-mRNA (36), respectively, the M10 protein also specifically inhibited export of the corresponding spliced RNAs (Figure 2C and D, and Supplementary Figure S1C and D). These results, taken together, clearly indicated that the Rev protein prevented RRE-containing spliced RNAs from utilizing the TAP-dependent export pathway.

### Rev inhibits the association of TAP with spliced ftzRRE RNA

Since the Rev protein suppressed TAP-dependent export of spliced RRE-containing RNAs, we next investigated whether the association of the mRNA export factor TAP with RNAs was affected by Rev. To this end, we performed GST-TAP231 pull-down experiments (43).

**Figure 3.**
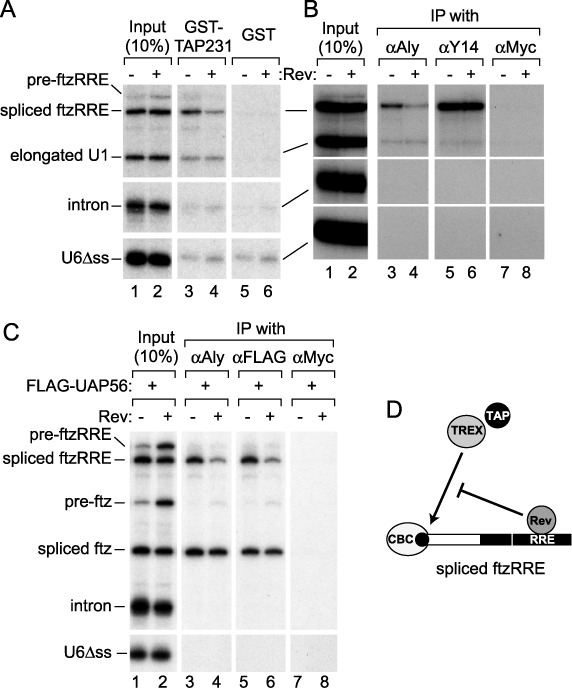
Effect of Rev on the association of mRNA binding proteins. (**A**) The same ^32^P-labeled RNA mixture as in Figure 1 was injected into the nucleus in the absence or presence of Rev. The nuclear fraction was prepared after 1 h, and GST pull-down was performed with glutathione beads that had been pre-bound with either the GST-TAP231 or GST protein. RNA precipitated with each type of bead was recovered and analyzed. The input lanes were loaded with 10% of each input mixture. (**B**) The nuclear fraction was prepared as in (A), and IP was performed with the anti-Aly/REF monoclonal antibody (11G5, αAly), anti-Y14 monoclonal antibody (4C4, αY14) or anti-Myc monoclonal antibody (9E10, αMyc) that had been pre-bound to Protein A-Sepharose beads. RNA precipitated with each antibody was recovered and analyzed. (**C**) The recombinant FLAG-UAP56 protein (50 fmol/oocyte) was pre-injected into the cytoplasm. After 16 h incubation, a second microinjection was performed into the nucleus with the same ^32^P-labeled RNA mixture as in Figure 1, except that pre-ftz RNA was used instead of elongated U1 RNA, in the absence or presence of Rev. IP was performed with 11G5, the anti-FLAG monoclonal antibody (M2, αFLAG), or 9E10. (**D**) Rev inhibits the association of TAP and the TREX complex with spliced ftzRRE RNA.

Both spliced ftzRRE and elongated U1 RNAs were specifically precipitated by GST-TAP231, as expected, in the absence of Rev, which indicated that these two RNAs were in a state that was capable of TAP association in the nucleus (Figure 3A, lane 3). In contrast, in the presence of Rev, the association ability was reduced by 70% with spliced ftzRRE RNA, but not with elongated U1 in this condition (Figure 3A, lane 4, and Supplementary Figure S2A for RNA binding of Rev). These results indicated that the Rev protein can specifically inhibit the association of TAP with spliced ftzRRE RNA.

### Rev inhibits the association of TREX components with spliced ftzRRE RNA

To understand how Rev inhibited the association of TAP with spliced RRE-containing RNAs, we next examined the effect of Rev on the association of Aly/REF with RNA. RNA IP experiments were performed either from the nuclear fraction of oocytes that had been injected with the same ^32^P-labeled RNAs as in Figure 3A (Figure 3B and C, and Supplementary Figure S2B) or from the *in vitro* splicing reaction mixture (Supplementary Figure S2C–E).

mRNA-specific binding proteins such as Aly/REF, Y14 and the bulk SR proteins were associated with spliced ftzRRE RNA and elongated U1 RNA in the oocytes (Figure 3B, lanes 3 and 5, and Supplementary Figure S2B), and co-injection of Rev only reduced the association of Aly/REF with spliced ftzRRE RNA (by 54%; Figure 3B, lanes 4 and 6, and Supplementary Figure S2B). Furthermore, antibodies against Aly/REF specifically precipitated spliced ftzRRE RNA from the *in vitro* splicing reaction with HeLa cell nuclear extracts, and adding the Rev protein to the reaction greatly reduced the association of Aly/REF (by 50% for 11G5 and by 75% for KJ58 (10); Supplementary Figure S2C–E). The association of Y14 was not affected by Rev (Supplementary Figure S2E).

UAP56 is another component of the TREX complex (3) and promotes the recruitment of Aly/REF onto both spliced and intronless mRNAs through their direct interaction (37,55,56). Co-injection of Rev reduced the association of FLAG-UAP56 and Aly/REF with spliced ftzRRE RNA but not spliced ftz RNA (by 50%; Figure 3C). These results clearly indicated that Rev specifically inhibited the association of Aly/REF and UAP56, possibly in the context of the TREX complex, with spliced ftzRRE RNA (Figure 3D).

### Rev does not interfere with EJC formation

**Figure 4.**
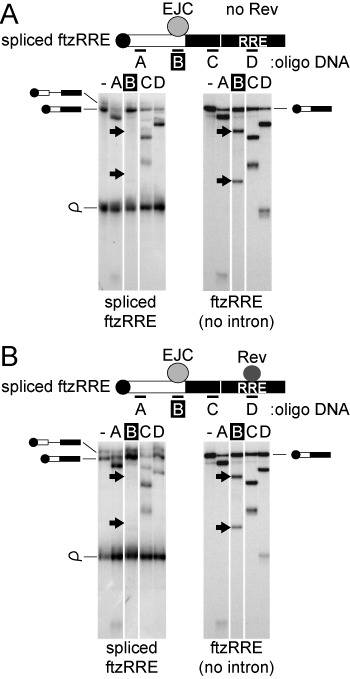
Effect of Rev on EJC formation. ^32^P-labeled pre-ftzRRE RNA (left) or intronless ftzRRE RNA (right) was microinjected into the nucleus in either the absence (**A**) or presence (**B**) of Rev. The nuclear fraction was prepared after 1 h, and RNase H digestion was performed with the antisense oligo DNAs A–D. RNA digestion was analyzed using 6% denaturing PAGE.

The splicing reaction deposits another large protein complex, the EJC, onto mRNAs, a component of which is Y14 (42,57). Because Rev did not inhibit the association of Y14 (Figure 3B and Supplementary Figure S2E), it was likely that Rev did not inhibit the formation of EJC on spliced ftzRRE RNA. To confirm this, we employed the RNase H protection assay originally used to map the location of EJC on spliced RNA (42). The presence of EJC should inhibit cleavage by RNase H with an antisense DNA oligo that hybridizes near the exon–exon junction. In the absence of Rev (Figure 4A), spliced ftzRRE RNA was efficiently cleaved by RNase H with various oligo DNAs, which hybridized to various regions of the spliced RNA, except with oligo B that hybridized near the exon–exon junction, confirming that EJC was properly formed near the exon–exon junction (Figure 4A, left), whereas control intronless ftzRRE RNA was cleaved with all the oligos (Figure 4A, right). Importantly, exactly the same results were obtained even in the presence of Rev (Figure 4B), which indicated that Rev did not affect the formation of EJC itself on spliced ftzRRE RNA. And the composition of EJC was not altered by Rev since the incorporation of Aly/REF as well as Y14 into EJC was not hindered by Rev (Supplementary Figure S3). It should be emphasized that the association of the TREX complex with RNA appears to be more important for RNA export (4,58).

### Rev inhibits the association of Aly/REF with intronless RRE-containing RNAs

**Figure 5.**
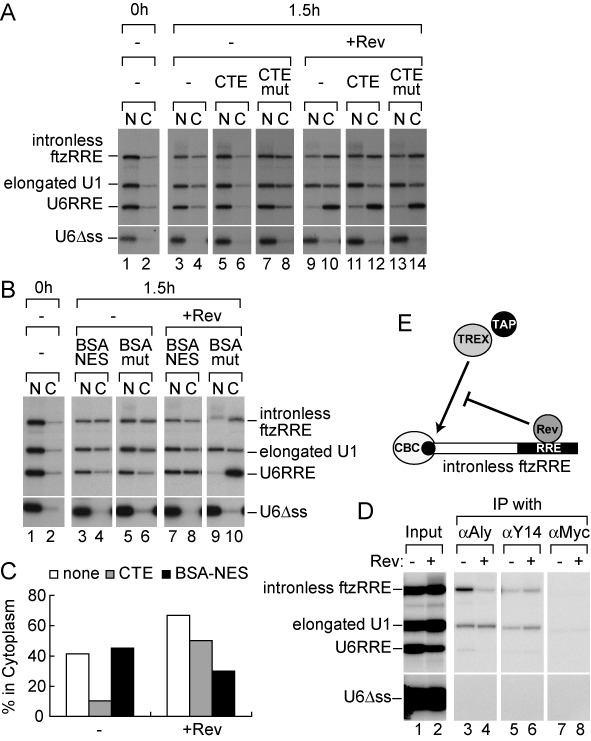
Effect of Rev on the export of intronless RNA containing RRE. (**A** and **B**) The same experiments as in Figure 1C and D, respectively, were performed, except that intronless ftzRRE RNA was used instead of pre-ftzRRE RNA and incubation was performed for 1.5 h. (**C**) Quantitation of the export of ftzRRE RNA in (A) and (B). (**D**) IP was performed as in Figure 3B, except that intronless ftzRRE RNA was used instead of pre-ftzRRE RNA. (**E**) Rev inhibits the association of TAP and the TREX complex with intronless ftzRRE RNA.

Since TAP and Aly/REF were also shown to be involved in the nuclear export of intronless mRNA (10,29,37,41,58,59), whether Rev could inhibit the association of Aly/REF with intronless mRNAs containing RRE was next examined (Figure 5). TAP-dependent export of intronless ftzRRE RNA was markedly suppressed by Rev, whereas TAP-dependent export of elongated U1 was hardly affected (Figure 5C for quantitation). This suppression of the TAP-dependent export pathway was likely due to the reduced association of Aly/REF with intronless ftzRRE RNA in the presence of Rev (Figure 5D and Supplementary Figure S2A for RNA binding of Rev). These results confirmed that Rev generally inhibited the association of Aly/REF with RRE-containing RNAs regardless of whether RNAs had been produced through splicing or not (Figures 3D and 5E). It is likely that this mechanism operates during the export of the unspliced HIV-1 transcript.

### The TAP-dependent pathway reduces singly spliced and unspliced HIV-1 RNA levels

**Figure 6.**
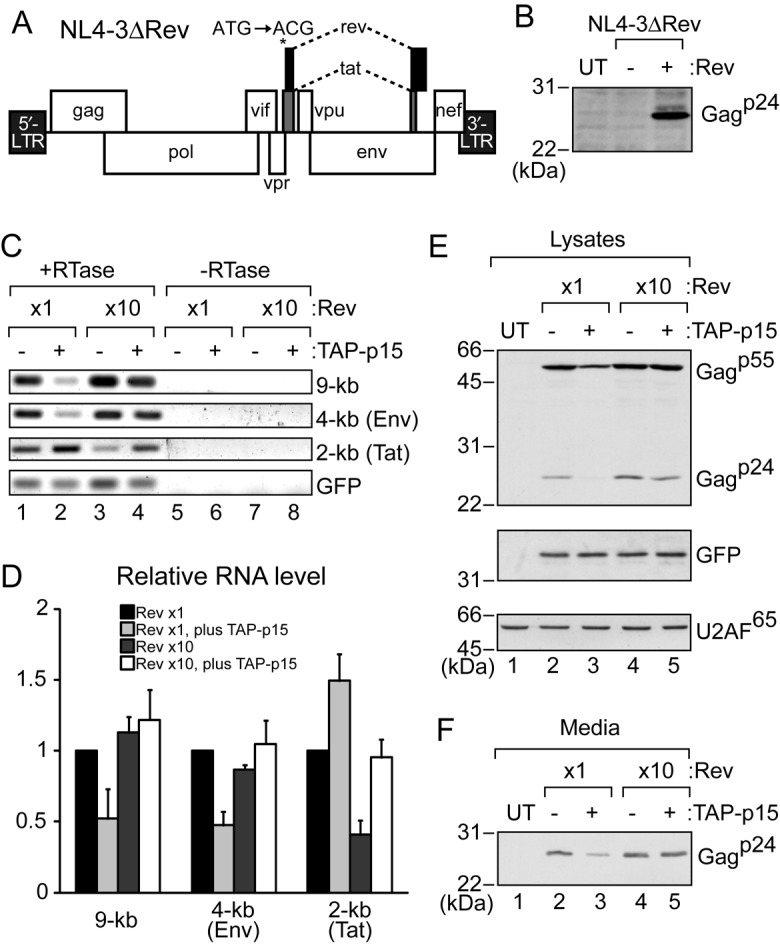
Effect of TAP-p15 overexpression on HIV-1 expression. (**A**) Genome organization of HIV-1 NL4-3ΔRev. The start codon of the Rev gene was mutated from ATG to ACG. (**B**) HEK293T cells in a 6-well plate (70% confluent) were transfected with pcDNA3-GFP (1 μg), pNL4-3ΔRev (1 μg), and either pCI-neo (0.1 μg) (−) or pCI-FLAG-Rev (0.1 μg) (+). After 24 h, cells were collected and proteins from cell pellets were analyzed by SDS-PAGE and western blotting with rabbit anti-Gag^p55^ antiserum. UT: untransfected cells were used as a control. (**C**) HEK293T cells in a 6-well plate (70% confluent) were transfected with pcDNA3-GFP (1 μg), pNL4-3ΔRev (1 μg), pCI-FLAG-Rev (0.1 or 1 μg), and either pcDNA5 (1 μg) (−) or pcDNA5-FLAG-TAP (0.6 μg) plus pcDNA5-FLAG-p15 (0.3 μg) (+). 0.9 μg of pCI-neo was added for 0.1 μg of pCI-FLAG-Rev to equalize the amount of plasmid DNAs. Supernatants and cells were collected after 24 h. RNA from cell pellets was subjected to semi-quantitative RT-PCR. PCR products were analyzed by electrophoresis in a 2% agarose gel. (**D**) Quantitation of the relative level of RNAs from three independent experiments performed as in (C). GFP mRNA was used for normalization. 0.1 μg of the pCI-FLAG-Rev sample (lane 1) was set to 1. Averages and standard deviations are shown. (**E**) Protein from the cell pellets in (C) was analyzed by SDS-PAGE and western blotting with rabbit anti-Gag^p55^ antiserum or the anti-GFP antibody. UT: untransfected cells were used as a control. U2AF^65^ was a loading control. (**F**) The filtrated media from (C) were immunoprecipitated with rabbit anti-Gag^p55^ antiserum and detected by western blotting with the monoclonal anti-Gag^p24^ antibody.

Why the TAP-dependent pathway had to be suppressed for singly spliced and unspliced transcripts by Rev remained unclear. To obtain a clue, we investigated what happened to HIV-1 gene expression when TAP was artificially overexpressed (Figure 6).

The HIV-1 DNA clone pNL4-3 derivative, pNL4-3ΔRev, was used for HIV-1 gene expression. pNL4-3ΔRev has a mutation (ATG to ACG) in the start codon of the Rev gene in pNL4-3, which is a silent mutation for the HIV-1 Tat gene (Figure 6A). pCMV-Rev was transfected into HEK293T cells concurrently with pNL4-3ΔRev to supply the Rev protein. The Rev protein was required for the expression of a translation product of the unspliced transcript, Gag^p24^ (Figure 6B and Supplementary Figure S4B), which demonstrated that this virus expression system worked well.

To examine the effect of TAP on virus expression, pNL4-3ΔRev was transfected with pCMV-Rev, pCMV-TAP, pCMV-p15 and pCMV-GFP as a control. When the TAP-p15 heterodimer was overexpressed by six times that of endogenous TAP (Supplementary Figure S4C), the level of singly spliced (4-kb, Env mRNA) and unspliced (9-kb) transcripts was significantly reduced, whereas that of the fully spliced (2-kb, Tat mRNA) transcript was not (Figure 6C, lanes 1 and 2, and D for quantitation), suggesting that an enhanced association of TAP to the intron-containing HIV-1 transcripts somehow reduced their expression levels. If Rev inhibits the association of TAP with RRE-containing RNAs in HEK293T cells, reduced RNA levels due to TAP-p15 overexpression may possibly be recovered by Rev overexpression. Rev co-overexpressed with TAP-p15 (Supplementary Figure S4C) indeed canceled this reduction (Figure 6C, lanes 1, 2 and 4, and D), which supported the model in which Rev inhibits the association of TAP with singly spliced and unspliced transcripts in HIV-1-expressing cells. However, Rev overexpression alone did not affect the levels of singly spliced and unspliced transcripts, but reduced the fully spliced transcript. The reason for the reduced levels of the fully spliced transcript is unknown. The reduction is likely unrelated to the splicing reaction because TAP-p15 overexpression reduced the transcripts in the presence of the splicing inhibitor, Spliceostatin A (SSA) (Supplementary Figure S4D) (60). Consistent with the level of the unspliced transcript, TAP-p15 overexpression reduced the cellular level of Gag^p55^, translated from the transcript, and Gag^p24^, processed from Gag^p55^, and Rev co-overexpression canceled this reduction (Figure 6E). Viral production was also likely suppressed by TAP-p15 overexpression, as demonstrated by the amounts of Gag^p24^ in the culture media (Figure 6F) (25). These results are consistent with the hypothesis that Rev suppresses the TAP pathway to avoid a reduction in the expression of singly spliced and unspliced transcripts that leads to reduced viral propagation.

We tried to investigate whether Rev inhibits the association of TAP with singly spliced and unspliced transcripts in HIV-1-expressing cells. However, we could not detect the association of TAP with the transcripts, regardless the Rev expression. This may be because TAP association destabilizes the transcripts.

### Molecular mechanism for Rev's remodeling of the composition of export RNPs

The above results show that Rev generally inhibits the association of the TREX complex with RRE-containing RNAs. However, the detailed mechanism for this was still unknown. RRE is located in the second intron of the HIV-1 primary transcript, while the TREX complex is recruited to a region near the 5′-terminus of mRNA because Aly/REF interacts with CBC. How distantly positioned Rev affected the association of the TREX complex remained to be elucidated. We speculated that Rev bound to RRE may interact with CBC bound to the cap of the same RNA, thereby competitively inhibiting the interaction between CBC and Aly/REF.

**Figure 7.**
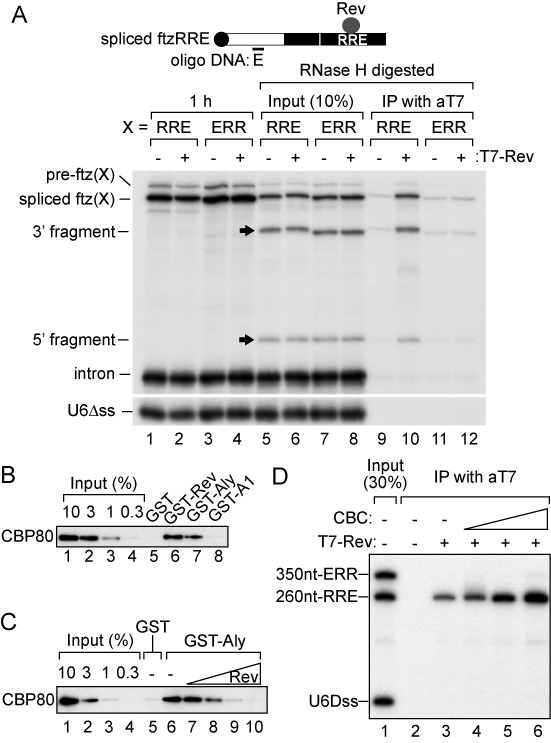
Molecular mechanism of remodeling of export RNPs by Rev. (**A**) ^32^P-labeled U6Δss RNA together with either pre-ftzRRE RNA (RRE) or pre-ftzERR RNA (ERR) was microinjected into the nucleus in either the absence or presence of Rev. The nuclear fraction was prepared after 1 h (lanes 1–4), and RNase H digestion was performed with the antisense oligo DNA E (lanes 5–8). The reaction mixture was immunoprecipitated with the anti-T7 antibody (lanes 9–12). RNAs were analyzed using 6% denaturing PAGE. (**B**) Purified recombinant CBP80 (3 μg) was pulled down by GST, GST-T7-Rev, GST-Aly or GST-hnRNP A1 (GST-A1) (1 μg each) in the presence of RNase A (1 mg/ml). Pulled down CBP80 was separated by SDS-PAGE and detected by western blotting. (**C**) Recombinant CBP80 (3 μg) was pulled down by GST or GST-Aly (1 μg each) in the presence of RNase A (1 mg/ml) and in the absence or presence of T7-Rev (0.2, 1, 5 or 25 μg), and was analyzed as in (B). (**D**) ^32^P-labeled U6Δss, m^7^G-capped 350 nt-ERR RNA and m^7^G-capped 260 nt-RRE RNA were incubated with or without recombinant T7-Rev (30 nM) or CBC (30, 100, 300 nM). The reaction mixture was immunoprecipitated with the anti-T7 antibody. Precipitated RNAs were analyzed by 8% denaturing PAGE.

To investigate Rev's association with the cap-proximal region, we performed RNA IP experiments from the nuclear fraction of *Xenopus* oocytes (Figure 7A). The nuclear lysate was prepared 1 h after the injection and was incubated with an antisense oligo E that hybridized with spliced ftzRRE as shown in Figure 7A, located between oligos A and B in Figure 4. This triggered digestion by endogenous RNase H to produce 5′ and 3′ fragments (Figure 7A, lanes 5–8). When purified recombinant T7-Rev protein was injected with RNAs, not only spliced ftzRRE RNA and the 3′ fragment, but also the 5′ fragment were precipitated by the anti-T7 antibody (Figure 7A, lanes 9 and 10). In contrast, no fragment was precipitated when control pre-ftzERR RNA was injected (Figure 7A, lanes 11 and 12). These results indicated that Rev bound to not only the RRE sequence, but also directly or indirectly to the region near the 5′-terminal cap structure of RRE-containing RNA.

**Figure 8.**
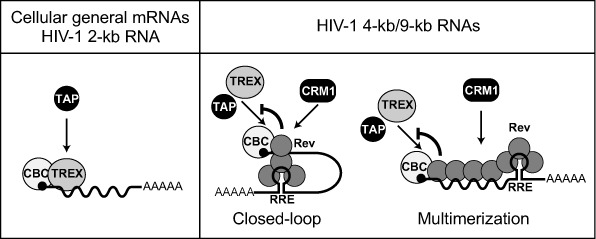
A model of how the HIV-1 Rev protein remodels viral export RNPs. Not all RNA binding proteins are shown for the sake of clarity. See the Discussion section for details.

How can Rev effectively associate with the cap-proximal region? We suspected an interaction between Rev and CBC. GST-Rev, but neither GST nor GST-hnRNP A1, pulled down CBP80, a component of CBC, in the presence of RNase A (Figure 7B, lane 6), which suggested that Rev directly interacted with CBC. The direct interaction between Aly/REF and CBP80 was verified (Figure 7B, lane 7) (4,5). Furthermore, we found that the interaction between CBP80 and Aly/REF decreased with the addition of Rev in a dose-dependent manner (Figure 7C), indicating that Rev competitively inhibited the interaction between CBC and Aly/REF. Although RNA was not present in this *in vitro* system, Rev should interact with CBC most effectively on the same RNA molecule *in vivo*. Recombinant CBC indeed stimulated the association of Rev with m^7^G-capped RRE RNA *in vitro* (Figure 7D). This stabilization of Rev's RRE-binding by CBC should contribute to the efficient interaction between Rev bound to RRE and CBC bound to the cap, which may be mediated by RNA's looping-out or Rev's multimerization on the entire RNA region (Figure 8, see the Discussion section). This model can also explain how Rev's inhibition is specific only to RNAs containing RRE, but not to RNAs without the element.

## DISCUSSION

### Model for Rev's suppression of the TAP-dependent pathway

Our model for the role of the HIV-1 Rev protein in suppressing TAP-dependent export of RRE-containing RNAs is shown in Figure 8. Once a sufficient amount of the Rev protein accumulated in the nucleus, Rev bound to RRE of singly spliced and unspliced transcripts. Rev interacted with CBC at the 5′-terminus of RNA (Figure 7). How was this interaction achieved? One possibility is that RRE-containing RNA may form a closed-loop RNP by a physical association between the 5′-terminus and RRE through the CBC–Rev interaction (Figure 8 middle), similar to that between the 5′-terminal and poly(A) tail in mRNA translation (61). Alternatively, Rev may multimerize on the entire region of RNA, not only on the RRE region as previously demonstrated (16) (Figure 8 right). This multimerization may be stimulated by CRM1-binding because it enhances the Rev–Rev interaction (62). CBC stabilized the association of Rev with the 5′ region of the transcripts (Figure 7D). This stabilization may enhance the multimerization of Rev. However, the fact that RNA is cleavable by DNA oligos and RNase H may favor the former model (Figures 4 and 7). In any case, the interaction between CBC and Rev inhibited the association of Aly/REF to the region near the 5′-termini of the transcripts through competitive inhibition of the interaction between CBC and Aly/REF (Figure 7C). Accordingly, the TAP-dependent pathway was inhibited and the CRM1-dependent pathway was induced.

Although our results shown here support the above model, additional mechanisms may be involved in this remodeling. Many RNA helicases have been reported to interact with Rev. Such RNA helicases, in cooperation with Rev, may actively remove Aly/REF from RNA. RNA helicase DDX3, which was shown to be important for HIV-1 RNA export (63), may be a good candidate. In addition to Aly/REF, some shuttling SR proteins are thought to function as redundant adaptors for TAP recruitment (43,64). In this regard, although the association of bulk SR proteins was not affected by the presence of Rev (Supplementary Figure S2B), the possibility that the association of some specific SR proteins may be hindered by Rev cannot be excluded.

### Biological significance for Rev's suppression of the TAP-dependent pathway

An important question is why it is necessary for HIV-1 to suppress the TAP-dependent RNA export pathway of singly spliced and unspliced transcripts. In this regard, we showed that TAP-p15 overexpression, which may lead to the enhanced association of TAP with RNA, specifically reduced the level of singly spliced and unspliced transcripts (Figure 6C and D. Thus, HIV-1 inhibited the association of TAP with RRE-containing RNAs by utilizing Rev, most likely in order to circumvent the TAP-mediated reduction in HIV-1 gene expression.

This finding raises a new question that needs to be solved: what is the mechanism of the TAP-mediated reduction of singly spliced and unspliced transcripts? One possible explanation is based on the feature that transcripts contain introns. TAP being associated with unspliced pre-mRNAs is unfavorable because TAP may aberrantly export pre-mRNAs to the cytoplasm, which may lead to their translation into harmful proteins. To prevent this, the cell may actively retain and splice pre-mRNAs that are bound by TAP. In this regard, CTE-containing, therefore TAP-bound, pre-mRNAs were shown to be retained in the nucleus and reduced by the NPC-associated protein TPR (24).

### Relationships to other viral systems

Human T-lymphotropic virus (HTLV) also belongs to a retrovirus family and utilizes the homologous mechanism to export viral RNAs by the Rex-RxRE system (Rev-RRE in HIV-1). It is interesting to determine whether Rex has the same activity as the HIV-1 Rev protein to suppress TAP-dependent export of viral RNAs. Here we found that Rex interacted directly or indirectly with CBP80 (Supplementary Figure S5). Targeting Rev/Rex's remodeling activity could introduce a novel useful anti-retroviral therapy for HIV and HTLV. Other viruses also take advantage of RNA export regulation for their gene expression (65). The Herpes Simplex Virus ICP27 protein interacts with Aly/REF and recruits it onto intronless viral mRNAs that are otherwise inefficient substrates for Aly/REF association, which induces TAP-dependent export of viral mRNAs. The Epstein–Barr virus EB2 protein also has very similar activity. These two viral proteins are the exact mirror images of the function of the HIV-1 Rev protein, which inhibits Aly/REF association with RNA. Therefore, it may be a commonly used strategy for viruses to control the recruitment of Aly/REF in order to regulate export of their own mRNAs.

### One RNA-one export pathway hypothesis

A plethora of evidence has already been obtained supporting the link between the RNA export pathway and nuclear/cytoplasmic fate of RNA (24–31). Therefore, more than one RNA export pathway for a single RNA molecule is thought to be problematic for export RNPs. One of the solutions is the mechanism that allows the recruitment of export factors for only one particular export pathway. We previously observed a similar RNP remodeling process prior to the export of cellular RNAs (29,41). If an RNA is committed to the mRNA export pathway, U snRNA-specific export factors such as PHAX, an adaptor between CBC and CRM1 (38), are not found on the RNA. Conversely, if an RNA is committed to the U snRNA export pathway, mRNA-specific export factors such as Aly/REF are not found on the RNA. Thus, recruiting mRNA-specific factors and U snRNA-specific factors is mutually exclusive (29,41). This RNP remodeling is achieved by a similar mechanism to that described here for HIV-1 transcripts (27). The nuclear RNA binding protein hnRNP C binds to mRNA and CBC. Therefore, the association of PHAX with mRNA is competitively inhibited. Interestingly, when PHAX was permitted to associate with mRNA by hnRNP C knockdown, the export of mRNA was inhibited (27). This finding also illustrates that the choice of RNA export pathway defines the RNA's fate. It is also worth noting that CBC-bound hnRNP C must be replaced by Aly/REF in the course of the formation of export-competent RNP (27). Specifically for m^7^G-capped RNA, CBC binding proteins, such as hnRNP C, Aly/REF and PHAX, serve as a landmark of the RNA state. In other words, the identity of the factor that interacts with cap-bound CBC and occupies the cap-proximal region determines the RNA export pathway. Owing to this mechanism, if RNA is committed to one pathway, the association of the export factors of other pathways is competitively prevented. Rev may favor viral gene expression by occupying the region and determining the RNA export pathway.

## SUPPLEMENTARY DATA

Supplementary Data are available at NAR Online.

SUPPLEMENTARY DATA
