# Supplementary material for: HIV-1 Rev protein specifies the viral RNA export pathway by suppressing TAP/NXF1 recruitment
Source: Nucleic Acids Res. 2014 Apr 20;42(10):6645–58. doi: 10.1093/nar/gku304 (PMC4041468; doi:10.1093/nar/gku304)
Supplement: SUPPLEMENTARY DATA [file supp_42_10_6645__index.html]

HIV-1 Rev protein specifies the viral RNA export pathway by suppressing TAP/NXF1 recruitment — HIV-1 Rev protein specifies the viral RNA export pathway by suppressing TAP/NXF1 recruitment — HIV-1 Rev protein specifies the viral RNA export pathway by suppressing TAP/NXF1 recruitment — SUPPLEMENTARY DATA 

# HIV-1 Rev protein specifies the viral RNA export pathway by suppressing TAP/NXF1 recruitment

## SUPPLEMENTARY DATA

**Files in this Data Supplement:**

- SUPPLEMENTARY DATA
