# Supplementary material for: HIV-1 Rev protein specifies the viral RNA export pathway by suppressing TAP/NXF1 recruitment
Source: Nucleic Acids Res. 2014 Apr 20;42(10):6645–58. doi: 10.1093/nar/gku304 (PMC4041468; doi:10.1093/nar/gku304)
Supplement: SUPPLEMENTARY DATA [file supp_gku304_nar-03397-y-2013-File010.pdf]

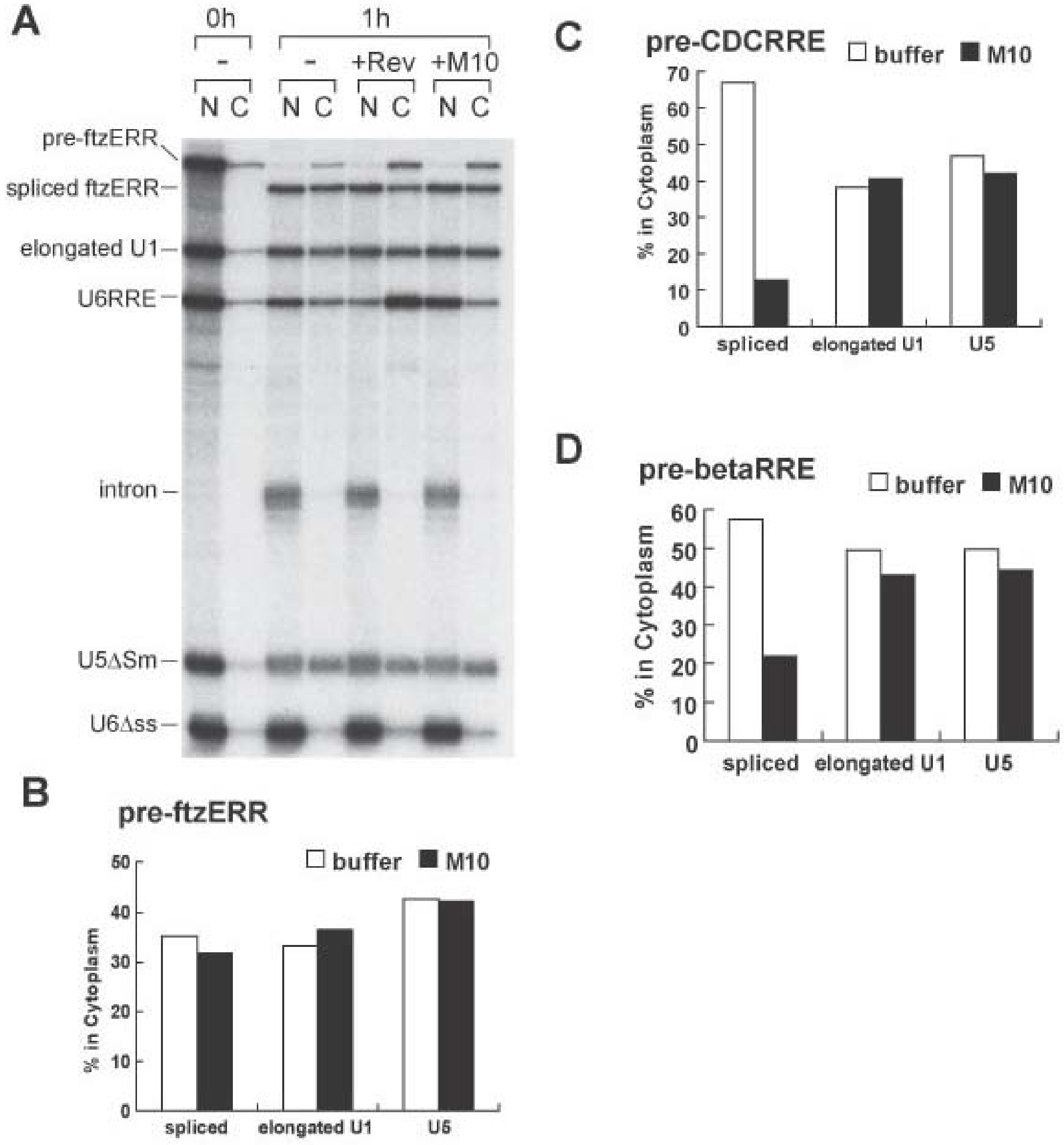

Supplementary Figure S1: Inhibition of TAP-dependent RNA export by Rev

(A) The same ^{32}P-labeled RNA mixture as in Figure 2A, except that pre-ftzERR, was microinjected into the nucleus. The effect of the wild-type Rev or Rev M10 mutant protein (160 fmol/oocyte) was examined as in Figure 2A. (B) Quantitation of the export of spliced ftzERR, elongated U1, and U5 RNAs is shown. (C and D) The same ^{32}P-labeled RNA mixture as in A, except that either pre-CDCRRE (C) or pre-betaRRE (D) was used instead of pre-ftzERR, was microinjected into the

nucleus. The effect of the wild-type Rev or Rev M10 mutant protein was examined as in A (Figure 2C and D). Quantitations of the results are shown. Effect of Rev M10 on the export of spliced CDCRRE (C) and spliced betaRRE (D).

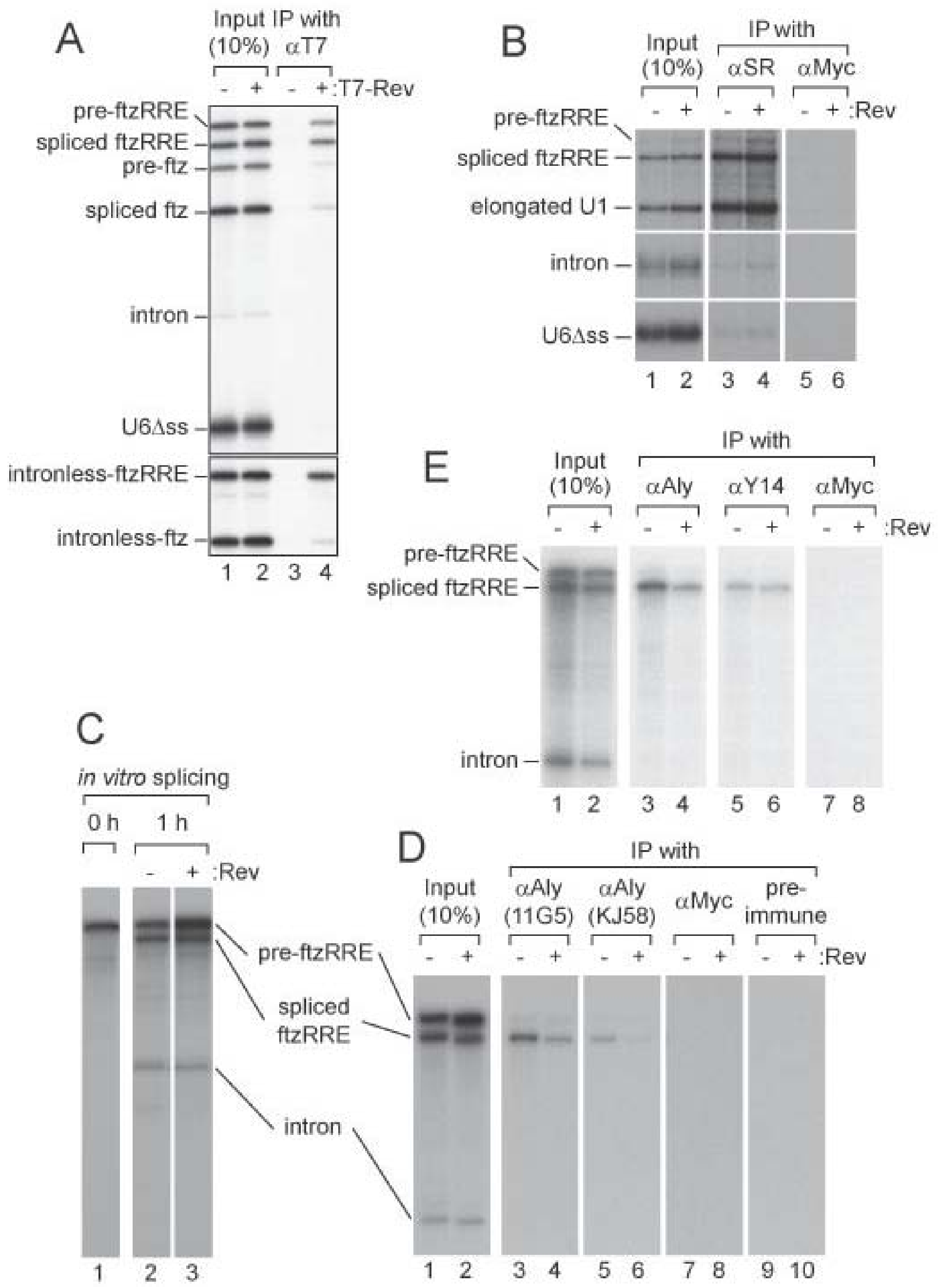

Supplementary Figure S2: Effect of Rev on the association of RNA binding proteins

(A) Rev bound to RRE-containing RNAs in Xenopus oocytes. ^{32}P-labeled pre-ftzRRE, pre-ftz, and U6Δss RNAs (upper panel) were microinjected into the nucleus in the absence or presence of T7-Rev. The nuclear fraction was prepared

after 1 hour, and IP was performed with the anti-T7 antibody that had been pre-bound to Protein A-Sepharose beads. ^{32}P-labeled intronless ftzRRE and intronless ftz RNAs (lower panel) were used in the same IP experiment.

(B) Effect of Rev on the association of SR proteins. The ^{32}P-labeled RNA mixture as was injected into the nucleus in the absence or presence of Rev. The nuclear fraction was prepared after 1 hour, and IP was performed with either the anti-SR monoclonal antibody 1H4 (αSR) or 9E10 (αMyc) that had been pre-bound to Protein A-Sepharose beads.

(C-E) Effect of Rev on the association of RNA binding proteins in *in vitro* splicing. ^{32}P-labeled pre-ftzRRE was subjected to *in vitro* splicing with HeLa cell nuclear extracts in the absence or presence of the recombinant Rev protein (2.5 μM) for 0 or 1 hour, and the RNA products were analyzed (C). The 1-hour reaction mixture prepared as in A was used for IP experiments as in Figure 3B (D). IP experiments with αAly (11G5), αY14 (4C4), or αMyc (9E10) were performed with the *in vitro* splicing reaction mixture as in B (E).

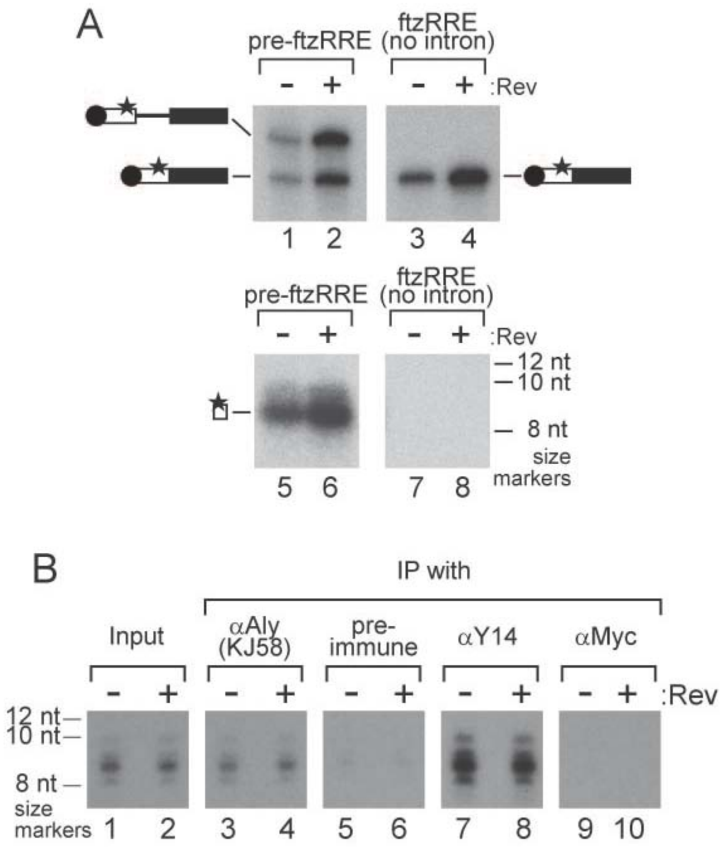

Supplementary Figure S3: Effect of Rev on the composition of EJC

(A) Pre-ftzRRE RNA (left) or intronless ftzRRE RNA (right) that had been singly labeled by ^{32}P near the exon-exon junction was subject to *in vitro* splicing with HeLa cell nuclear extracts in the absence or presence of the recombinant Rev protein (2.5 μM) for 1 hour, and RNA was analyzed without RNase digestion (lanes 1-4). Separately, the splicing reaction mixture was subjected to digestion with a mixture of RNase A and RNase T1 and protected RNA was analyzed using 20% denaturing PAGE (lanes 5-8). (B) The RNase-digested samples from A were subjected to IP with either the anti-Aly/REF polyclonal antibody (KJ58, αAly), anti-Y14 monoclonal antibody (4C4, αY14), pre-immune polyclonal antibody (preimmune), or anti-Myc monoclonal antibody (9E10, αMyc). The protected RNA fragment precipitated with each antibody was recovered and analyzed.

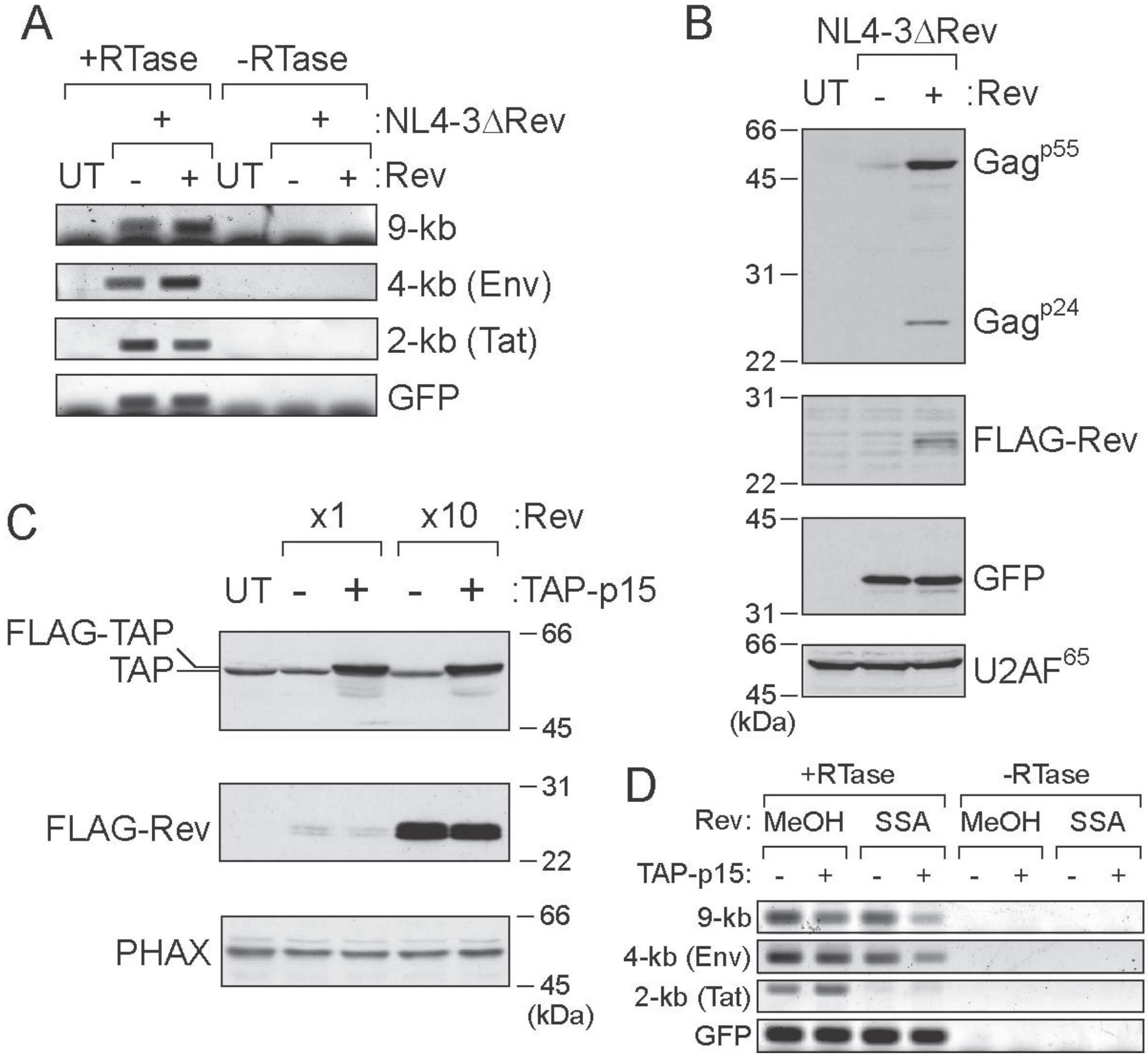

Supplementary Figure S4: Expression of viral RNAs and proteins

(A) RNA from cell pellets in Figure 6B was subjected to semi-quantitative RT-PCR. PCR products were analyzed by electrophoresis in a 2% agarose gel. UT: untransfected cells were used as a control. (B) Protein from cell pellets in Figure 6B was analyzed by SDS-PAGE and Western blotting with rabbit anti-Gag^{p55} antiserum, and anti-FLAG or anti-GFP antibodies. U2AF^{65} was a loading control. (C) TAP and FLAG-Rev proteins from the cell pellets in Figure 6C were analyzed by SDS-PAGE and Western blotting. PHAX was a loading control. (D) HEK293T cells in a 6-well

plate (70% confluent) were transfected with pcDNA3-GFP (1 μg), pNL4-3ΔRev (1 μg), pCDNA3-Rev (1 μg), and either pcDNA5 (1 μg) (-) or pcDNA5-FLAG-TAP (0.6 μg) plus pcDNA5-FLAG-p15 (0.3 μg) (+). After 6 h, SSA (100 ng/mL) was added. Cells were collected after 12 h. RNA from cell pellets was subjected to semi-quantitative RT-PCR. PCR products were analyzed by electrophoresis in a 2% agarose gel.

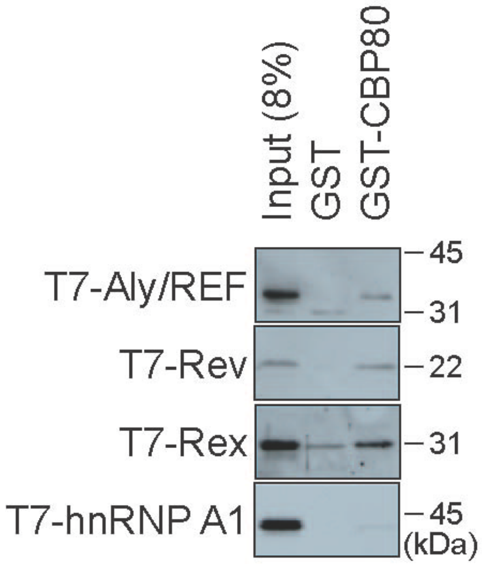

Supplementary Figure S5: Interaction between CBP80 and RNA binding proteins

T7 tagged-Aly/REF, Rev, Rex, and hnRNP A1 were individually produced in the Rabbit Reticulocyte Lysate TNT system (Promega). Each protein was pulled down by 1 μg of GST or GST-CBP80 in the presence of 0.5 mg/mL RNase A. Proteins were analyzed by SDS-PAGE and Western blotting with anti-T7 antibody.
